# A machine learning approach for early identification of patients with severe imported malaria

**DOI:** 10.1186/s12936-024-04869-3

**Published:** 2024-02-13

**Authors:** Alessandra D’Abramo, Francesco Rinaldi, Serena Vita, Riccardo Mazzieri, Angela Corpolongo, Claudia Palazzolo, Tommaso Ascoli Bartoli, Francesca Faraglia, Maria Letizia Giancola, Enrico Girardi, Emanuele Nicastri

**Affiliations:** 1 National Institute for Infectious Diseases “Lazzaro Spallanzani” IRCCS, Via Portuense 292, 00149 Rome, Italy; 2 Department of Mathematics “Tullio Levi-Civita”, University of Padova, Via Trieste, 63, 35131 Padua, Italy; 3 Department of Information Engineering, University of Padova, Via Giovanni Gradenigo, 6B, 35131 Padua, Italy

**Keywords:** Imported malaria, Machine learning, Severe malaria, Risk factors

## Abstract

**Background:**

The aim of this study is to design ad hoc malaria learning (ML) approaches to predict clinical outcome in all patients with imported malaria and, therefore, to identify the best clinical setting.

**Methods:**

This is a single-centre cross-sectional study, patients with confirmed malaria, consecutively hospitalized to the Lazzaro Spallanzani National Institute for Infectious Diseases, Rome, Italy from January 2007 to December 2020, were recruited. Different ML approaches were used to perform the analysis of this dataset: support vector machines, random forests, feature selection approaches and clustering analysis.

**Results:**

A total of 259 patients with malaria were enrolled, 89.5% patients were male with a median age of 39 y/o. In 78.3% cases, *Plasmodium falciparum* was found. The patients were classified as severe malaria in 111 cases. From ML analyses, four parameters, AST, platelet count, total bilirubin and parasitaemia, are associated to a negative outcome. Interestingly, two of them, aminotransferase and platelet are not included in the current list of World Health Organization (WHO) criteria for defining severe malaria.

**Conclusion:**

In conclusion, the application of ML algorithms as a decision support tool could enable the clinicians to predict the clinical outcome of patients with malaria and consequently to optimize and personalize clinical allocation and treatment.

**Supplementary Information:**

The online version contains supplementary material available at 10.1186/s12936-024-04869-3.

## Background

Malaria is currently a major clinical and epidemiological problem in the world, including European countries. In Europe, approximately 6000 imported malaria cases are reported annually, with 10% of them progressing towards severe malaria [1]. The risk of progression to severe malaria with multi-organ involvement and finally death once people are infected is very high and an early and prompt identification of patients with a poor prognosis is a challenge [2]. In the last decade, new mathematical approaches were used in medicine in order to solve health-related problems. More specifically, Machine Learning (ML) algorithms, which help building systems (i.e., mathematical models) able to learn information from a given sets of data, have recently become significant medical decision support tools. ML uses datasets to recognize complex connections between several patient characteristics, make predictions and provide personalized treatment [3]. ML approaches represent a new frontier in medicine and in the infectious disease field [4]. Specifically, ML methods were applied in malaria setting investigating various items ranging from immunological aspects to diagnostic tools and therapeutic options [5]. The aim of this study is to design ad hoc ML approaches to predict clinical outcome in all patients with imported malaria and, therefore, to identify the best clinical setting.

## Methods

### Design and participants

In this single-centre cross-sectional study, a total number of 259 patients with confirmed malaria consecutively hospitalized to the Lazzaro Spallanzani National Institute for Infectious Diseases, Rome, Italy, from January 2007 to December 2020 were retrospectively recruited. Inclusion criteria: age > 18 years old, written informed consent at hospital admission from the patient or next of kin if patient unable, confirmed malaria diagnosis with microscope parasite identification in the blood smear. Severe malaria was diagnosed according to the World Health Organization (WHO) malaria guideline [6]. For *Plasmodium vivax*, all the criteria were applied with the only exception of hyperparasitaemia. Demographic characteristics, medical and travel history, clinical presentation, anti-malarial and supportive treatment, parasitaemia before and during treatment, complications during treatment, adverse drug reactions, clinical outcome (survival, death or sequelae) at day 28 post-treatment were collected for all patients from the clinical record. In addition, the time to reduce parasite density below 1% and parasite clearance were also collected.

### Machine learning approaches

Thirty-two clinical and laboratory features were used to describe every patient in the dataset. These features were divided into three main categories, reported in Table 1.

**Table 1:** List of the features

Personal Features	Age, sex, comorbidity, European citizen, immigrant, visitor from endemic country, country of infection, previous malaria episode, duration of travel, medical care delay, diagnosis delay, chemoprophylaxis use, compliance to chemoprophylaxis use [13]
WHO criteria	Cerebral malaria/coma, convulsions, acute renal failure, respiratory failure, hypoglycaemia, shock, spontaneous bleeding, acidosis, jaundice, anaemia, hyperparasitaemia [11]
Blood values at admission	Baseline parasitaemia, platelet count, haemoglobin level, creatinine, total bilirubin, aspartate aminotransferase, sodium [7]

**Fig. 1:**
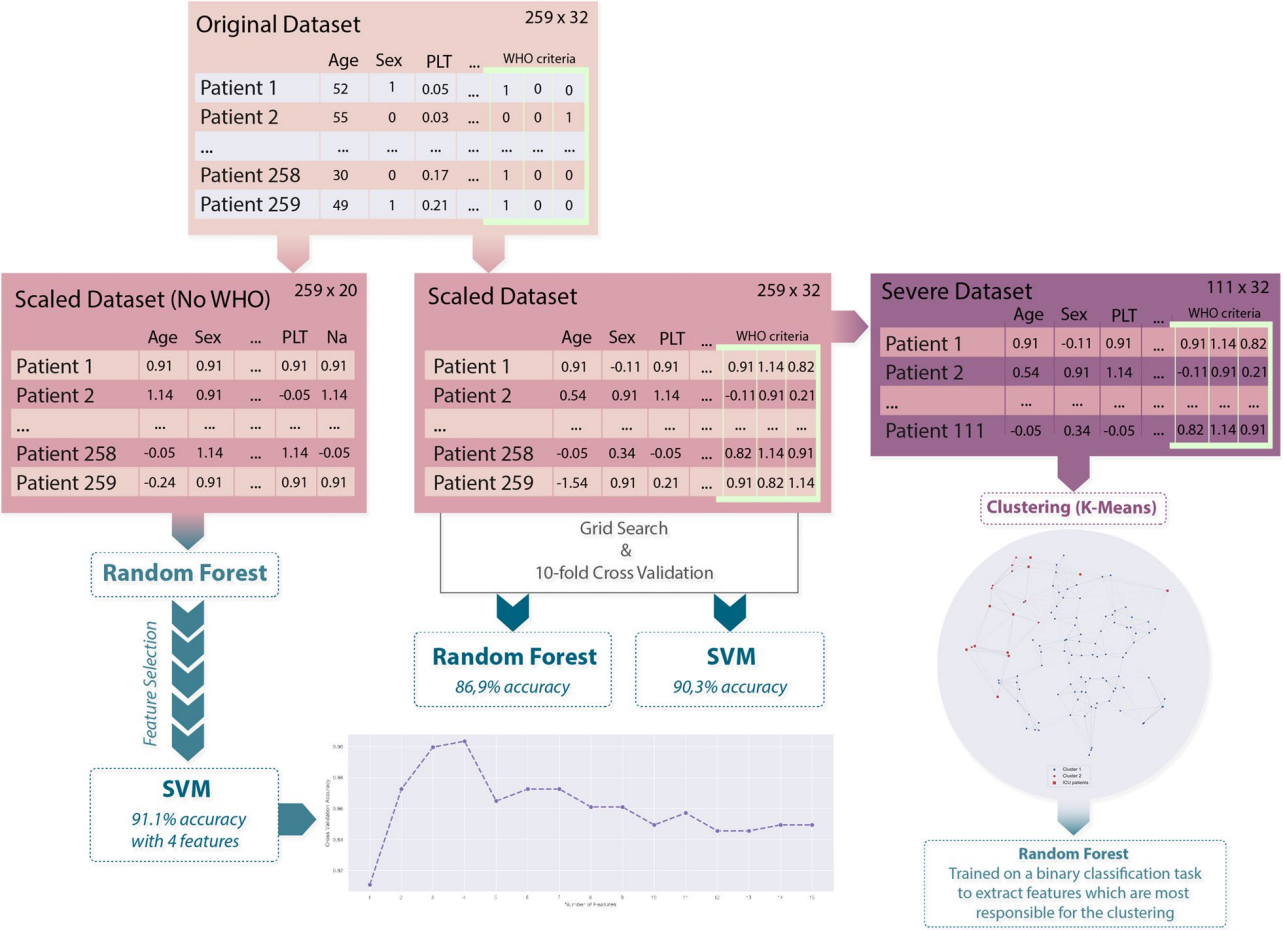
Machine learning workflow

Different ML approaches were used to perform the analysis of this dataset, more specifically: support vector machines, random forests, feature selection approaches and clustering analysis. A complete workflow related to the machine learning analysis is reported in Fig. 1.

In the first part of the study, two ML models were trained on the complete set of features. In this phase, the goal was to build a classifier to distinguish between patients with ‘severe malaria’ and ‘non-severe malaria’ (binary classification task). Firstly, support Vector Machines was considered in the analysis. These are very popular supervised ML models, used for both regression and classification problems in many different fields. Their good generalization abilities and robustness against overfitting make them a suitable choice for our problem. The second model class used was random forests [8] a very popular ensemble learning technique. Indeed, as the name suggests, those models rely on the predictions of multiple decision tree models, trained on different subsets of the available data. They inherit all the intrinsic advantages of such models, i.e., the interpretability of results and the ability to identify the most useful features for solving the task at hand. Random forest model was also used as an embedded feature selection method to filter the most important features in a later phase of the analysis. For both models, hyper-parameter optimization was performed via grid-search. To show that the models do not overfit the training data, results were reported in terms of K-fold Cross Validation accuracy (with k = 10). With this procedure each model was trained 10 different times over different training splits of the whole dataset, and evaluate the resulting accuracy on each of the corresponding test splits. The final reported CV accuracy will be the average of all the obtained test accuracies for each split. The same models were also trained on a subset of the dataset not containing the WHO features. This was done in order to evaluate the impact such features have on the decision process.

## Results

### Study population

From 2007 to 2020 a total of 259 patients with malaria were enrolled, 232 (89.5%) patients were male with a median age of 39 years old (IQR 29–71) (Table 2). Comorbidities were observed in 48 (18.5%) cases, 174 (67.1%) patients came from West Africa and 244 (94.2%) patients did not take any anti-malaria chemoprophylaxis. The median time of delay in diagnosis was 2 days (IQR 1–33). In 203 (78.3%) cases, *Plasmodium falciparum* was found. The patients were classified as severe malaria in 111 (42.8%) cases with a 2%-median baseline parasitaemia (IQR 1–27); of them 85/111 (76.5%) met ≤ 2 WHO criteria of severe malaria. Forty-two severe malaria patients with only 1 WHO criterion (37.8%) were treated with an oral anti-malarial drug. Twelve patients were admitted in Intensive care unit (ICU). All patients had a favourable clinical outcome.

**Table 2:** Study population: clinical features

Variables	Study Population (259)	Severe Malaria (n = 111)	Uncomplicated Malaria (n = 148)	*p*
Male (n, %)	189 (72.9%)	88 (79.2)	101 (68.2)	*ns*
Age (Median, IQR)	39 (29–71)	39 (29–71)	41 (29–70)	*ns*
Country of origin (n, %)				
European	107 (41.4)	55 (49.6)	52 (49)	
Immigrant	140 (54)	52 (46.8)	88 (46.5)	
Visitors from endemic country	12 (4.6)	4 (3.6)	8 (4.5)	*ns*
Comorbidities (n, %)	48 (18.5%)	26 (23.4%)	22 (14.8)	
Antimalarial chemoprophylaxis (n, %)	244 (94.2%)	9 (8.1)	6 (4)	*ns*
Area of infection (n, %): West Africa	174 (67.1%)	86 (77.4)	88 (59.4)	*ns*
Purpose of travel (n, %)				
VRFs	83 (32%)	36 (32.4)	47 (31.7)	
Tourism	47 (18.2%)	31 (27.9)	16 (10.8)	
Business	53 (20.4%)	22 (19.8)	31 (20.9)	
Humanitarian aid	28 (10.8%)	13 (11.8)	15 (10.2)	
Other	48 (18.6)	9 (8.1)	39 (26.4)	*ns*
*Plasmodium* (n, %)				
*Falciparum*	203 (78.3%)	102 (91.9)	101 (68.3)	
*Vivax*	38 (14.7%)	6 (5.4)	32 (21.7)	
*Ovale*	10 (3.9%)	1 (0.9)	9 (6)	
*Malariae*	3 (1.2%)	0	3 (2)	
*Knowlesi*	0	0	0	
*Mixed Infection*	5 (1.9)	2 (1.8)	3 (2)	*ns*
Delay of diagnosis (Median, IQR) (days)	2 (1–33)	2 (1–33)	2 (1–30)	*ns*
Delay of treatment (Median, IQR) (days)	3 (2–33)	3 (2–33)	3 (2–31)	*ns*
Days of hospitalization (Median, IQR) (days)	5 (4–75)	7 (5–75)	5 (4–19)	*0.001*
Basal % parasitaemia (Median, IQR)(days)	3 ± 2.7	5 ± 3	2 ± 1	*ns*
Time to parasitaemia < 1% (hours) (M ± SD)	37 ± 22.7	50 ± 27	28 ± 22	*0.004*
Time to parasite clearance (hours) (M ± SD)	58 ± 39.2	84 ± 45	39 ± 11	*0.005*
Antimalarial treatment				
Artesunate (iv)	36 (13.9)	25 (22.5)	11 (7.5)	*ns*
Quinine (iv)	26 (10)	25 (22.5)	1 (0.7)	
Artemetehr	19 (7.3)	12 (10.8)	7 (4.7)	
Quinine (oral)	20 (7.7)	14 (12.6)	6 (4.1)	
Dihydroartemisinin/piperaquine	125 (48.3)	32 (28.9)	93 (62.8)	
Chloroquine	33 (12.8)	3 (2.7)	30 (20.2)	

*ICU*  intensive care unit, *im* intramuscular, *iv* intraveneous, *M*  mean, *SD*  standard deviation, *IQR* interquartile range, *VRFs*  visiting relatives and friends

### Machine learning

The final results obtained by our ML models were reported in the Table 3:

**Table 3:** Support Vector Machine and Random forest CV accuracy

	Support Vector Machine (RBF) CV accuracy	Random Forest CV accuracy
With WHO criteria	90.3%	86.9%
With no WHO criteria	81.9%	84.6%

**Fig. 2:**
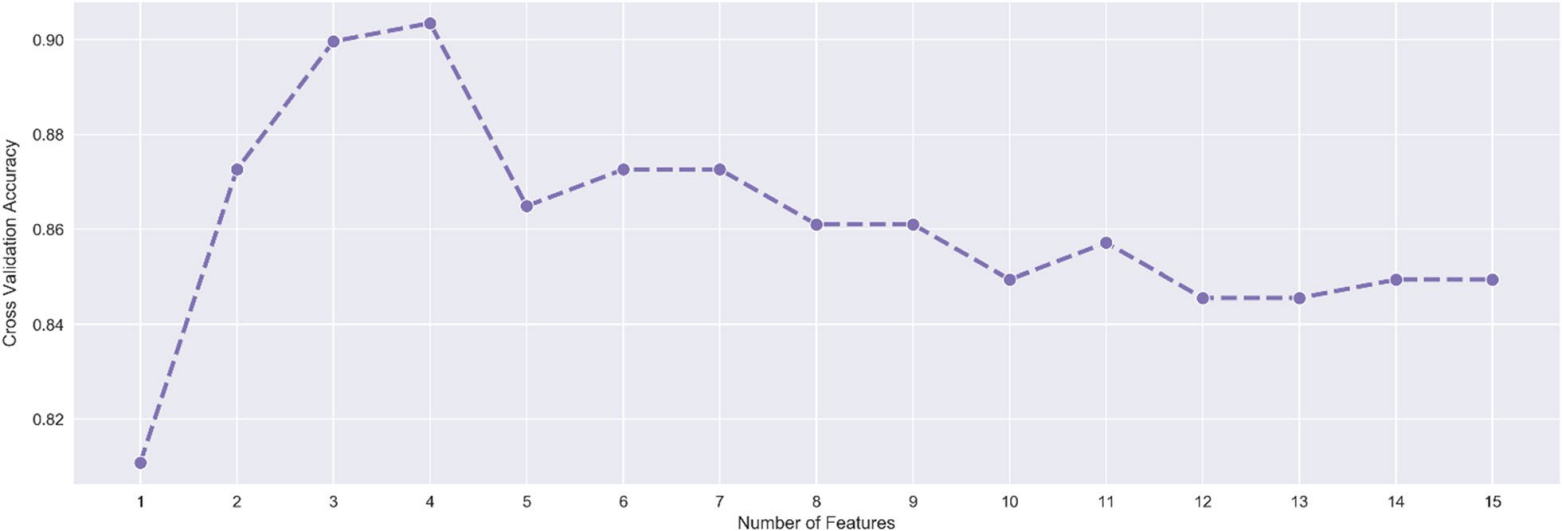
Cross validation accuracy obtained with increasing number of features

Subsequently, a feature selection method based on random forests was applied to select the most relevant features in the considered classification task. Indeed, considering the whole set of features in the training phase might not always lead to higher accuracies: irrelevant or redundant information might be introduced, hindering the generalization capabilities of the classifier and increasing the computational cost for training. The scores assigned to the features by random forest led to interesting results: using only the first 4 most important features (not included in the WHO criteria), SVM managed to reach an accuracy of 91.1% (Fig. 2).

Such features are:

1. Baseline parasitaemia;
2. Total bilirubin;
3. Aspartate aminotransferase (AST);
4. Platelet count.

In the last phase of the analysis, the goal was to identify the most important features related to the severe malaria patients admitted in ICU (11 samples out of 111). The problem was addressed by means of an unsupervised learning technique: the K-Means clustering method (with the Euclidean metric). Note that, unlike before, by employing an unsupervised ML technique, for structure inherently present in the patient features were searched, without providing ground truth labels to the learning procedure. A two-dimensional network visualization of the clusters was obtained using t-SNE [9] (Additional file 1). Interesting results were found using the whole set of features: two clusters were identified by the algorithm. The smaller one, composed by 19 patients included all the unfavourable outcomes (i.e., the 11 ICU patients) and 8 patients that were subject to prolonged hospitalization due to some other complications (e.g., comorbidities, bacterial infections). Once again, random forest based feature selection techniques were employed to understand the most important features characterizing the clusters: in this case the main role was played by the AST value and by two of the WHO criteria (renal failure and respiratory failure). Summarizing, four parameters, AST, platelet count, total bilirubin and parasitaemia, could be considered in the identification and evaluation of a negative outcome. Interestingly, two of them, aminotransferase and platelet are not included in the current list of WHO criteria for defining severe malaria. Furthermore, the consistency of the cluster analysis for severe malaria cases was confirmed by the evidence that all the 19 patients included in the smaller group were subject to prolonged hospitalization due to complications related to background comorbidities, bacterial infections and/or ICU admissions. In this cluster, apart from baseline AST, acute renal and respiratory failure, already included in the list of current WHO criteria, were strongly associated to the negative clinical outcome.

## Discussion

Malaria remains a substantial problem in non-endemic countries where represents a medical emergency. Severe malaria may rapidly evolve to an unfavourable prognosis with a case-fatality rate between 5 and 10% [6]. Unspecific and overlapping symptoms lead to a delayed access to care, diagnosis and initiation of specific therapy. In this cohort, 111 pts had severe malaria and 12 patients required ICU care, with a 3-day median delay of malaria diagnosis; most of them had been infected in West Africa, none of them received anti-malarial chemoprophylaxis and *P. falciparum* was the main causing species. Several studies have been published with the aim of identifying predictive factors of disease severity. In the 400-patient French malaria cohort, three baseline variables independently predicted death: older age, coma and high parasite density [10]. In a previous study, an early assessment of the severity status of the patient by specific score was required at admission to rapidly drive correct patient admission in critical care area. Applying both malaria-specific (Glasgow coma scale, Creatinine, Respiratory rate, Bilirubin, Systolic blood pressure, GCRBS) and general (System Organ Failure Assessment, SOFA) scores to severe malaria patients, could be the best approach to assess the need for intensive care. Finally, the number of WHO criteria and AST plasma level can predict the need of intensive care [11]. Recently, the use of machine learning to solve health related problem is a new challenge. In particular, in the field of infectious disease, the applicability of expert approaches could support physicians to improve diagnosis and specific syndromic approach considering that the standard clinical management may not be fully appropriate. In malaria setting, the use of ML seems to be promising. Previously, ML methods have been applied in malaria setting to investigate various items ranging from immunological aspects to diagnostic tools and therapeutic options. In 2018, Kalantar-Motamedi et al*.* proposed a combined transcriptional drug repositioning/discovery and ML methods in order to identify new therapeutic synergistic drug combinations [12]. Bernabeu et al. revealed the interplay between cellular and molecular determinants, parasite biomass and clinical disease severity, through ML analysis [13]. Cominetti et al. using a network-based clustering method, revealed a strong correlation between disease heterogeneity and mortality using the current WHO definition in a population of 2915 Gambian children with malaria [14]. In this study, different ML approaches were used to perform the analysis of the considered dataset, more specifically: support vector machines, random forests, feature selection approaches and clustering analysis. Four baseline parameters, AST, platelet count, total bilirubin and parasitaemia, were all independently associated to an unfavourable outcome. The WHO does not consider transaminases and platelet dysfunctions as criteria for severe malaria definition due to the variable and non-specific nature of these parameters. Their disbalances may occur in several communicable or non-communicable diseases and are not exclusively reported in severe malaria cases. During malaria infection, at liver stage, sporozoites invade the hepatocytes which can cause organ congestion, sinusoidal blockage, and cellular inflammation; hepatocyte injury due to malaria runs elevated AST and ALT serum level enzymes [15]. Indeed, thrombocytopenia seems to occur primarily by peripheral destruction, bone marrow disjunctions, increased spleen sequestration and removal, consumption by disseminated intravascular coagulopathy, and, finally, clumping of *Plasmodium*-infected erythrocytes [16, 17]. Although these two parameters (AST and platelets) are widely recognized as markers of severe malaria there is no solid evidence to include its in the severe malaria definition. This study, indeed, suffers of a similar limitation: it has a retrospective design, has been conducted in a single centre, and a limited despite extensive follow-up data collection period even. However, the consistency of the cluster analysis among severe cases was confirmed by the evidence that all the 19 patients who clustered in the smallest group had a prolonged hospital stay, which was complicated by exacerbations of background comorbidities, occurrence of bacterial infections and/or ICU admissions. In this cluster, apart from baseline AST, acute renal and respiratory failures, already included in the list of current WHO criteria, were strongly associated to unfavourable outcome.

## Conclusion

In this study, the ML analysis identified unknown parameters associated with severe malaria, easily obtained from routinely laboratory tests. In conclusion, the application of ML algorithms as a decision support tool could enable the clinicians to predict the clinical outcome of patients with malaria and consequently to optimize and personalize clinical allocation and treatment.

### Supplementary Information

**Additional file 1: Fig. S1.** A visualization of the clusters related to the severe malaria patients, obtained using K-means.

## Data Availability

All data generated or analysed during this study are included in this published article.

## Abbreviations

ML: Machine learning
WHO: World Health Organization
IQR: Interquartile range
ICU: Intensive care unit
SVMs: Support vector machines
RBF: Radial basis function
CV: Cross Validation
AST: Aspartate Aminotransferase
GCRBS: Glasgow coma scale, Creatinine, Respiratory rate, Bilirubin, Systolic blood pressure
SOFA: System Organ Failure Assessment
im: Intramuscular
iv: Intraveneous
M: Mean
SD: Standard deviation
VRFs: Visiting relatives and friends

## References

[1] European Centre for Disease Prevention and Control. Annual epidemiological report 2014-emerging and vector-borne disease. Stockholm: ECDC; 2014.

[2] Greenberg AE, Lobel HO (1990). Mortality from *Plasmodium falciparum* malaria in travelers from the United States, 1959 to 1987. Ann Intern Med.

[3] Rajkomar A, Jeffrey D, Kohane I (2019). Machine learning in medicine. N Engl J Med.

[4] Valleron AJ (2017). Data science priorities for a university hospital-based institute of infectious diseases: a viewpoint. Clin Infect Dis.

[5] Schwalbe N, Wahl B (2020). Artificial intelligence and the future of global health. Lancet.

[6] WHO. World malaria report 2023. Geneva, World Health Organization, 2023.

[7] Cortes C, Vapnik V (1995). Support-vector network. Mach Learn.

[8] Breiman L (2001). Random forest. Mach Learn.

[9] Van deer Maaten L, Hinton G (2008). Visualizing data using t-SNE. J Mach Learn Res.

[10] Bruneel F, Tubach F, Corne P, Megarbane B, Mira JP, Peytel E, et al. Severe imported malaria in adults (SIMA) study group Severe imported falciparum malaria: a cohort study in 400 critically ill adults. PLoS One. 2010;5:e13236. 10.1371/journal.pone.0013236 PMC2951913 20949045

[11] D’Abramo A, Lepore L, Iannetta M, Gebremeskel Tekle S, Corpolongo A, Scorzolini L, Spallanzani Group for Malaria Study (2019). Imported severe malaria and risk factors for intensive care: a single-centre retrospective analysis. PLoS ONE.

[12] Kalantar-Motamed Y, Eastman RT, Guha R, Bender A (2018). A systematic and prospectively validated approach for identifying synergistic drug combinations against malaria. Malar J.

[13] Bernabeu M, Danziger SA, Avril M, Vaz M, Babar PH, Brazier AJ (2016). Severe adult malaria is associated with specific PfEMP1 adhesion types and high parasite biomass. Proc Natl Acad Sci USA.

[14] Cominetti O, Smith D, Hoffman F, Jallow M, Thézénas ML, Huang H (2018). Identification of a novel clinical phenotype of severe malaria using a network-based clustering approach. Sci Rep.

[15] Megabiaw F, Eshetu T, Kassahun Z, Aemero M (2022). Liver enzymes and lipid profile of malaria patients before and after antimalarial drug treatment at Dembia Primary Hospital and Teda Health Center, Northwest, Ethiopia. Res Rep Trop Med.

[16] Dos-Santos JCK, Silva-Filho JL, Judice CC, Kayano ACAV, Aliberti J, Khouri R (2020). Platelet disturbances correlate with endothelial cell activation in uncomplicated *Plasmodium vivax* malaria. PLoS Negl Trop Dis.

[17] Punnath K, Dayanand KK, Chandrashekar VN, Achur RN, Kakkilaya SB, Ghosh SK (2019). Association between inflammatory cytokine levels and thrombocytopenia during *Plasmodium falciparum* and *P. vivax* infections in South-Western Coastal Region of India. Malar Res Treat..

