# Supplementary figures and images for: A machine learning approach for early identification of patients with severe imported malaria
Source: Malar J. 2024 Feb 13;23:46. doi: 10.1186/s12936-024-04869-3 (PMC10865572; doi:10.1186/s12936-024-04869-3)

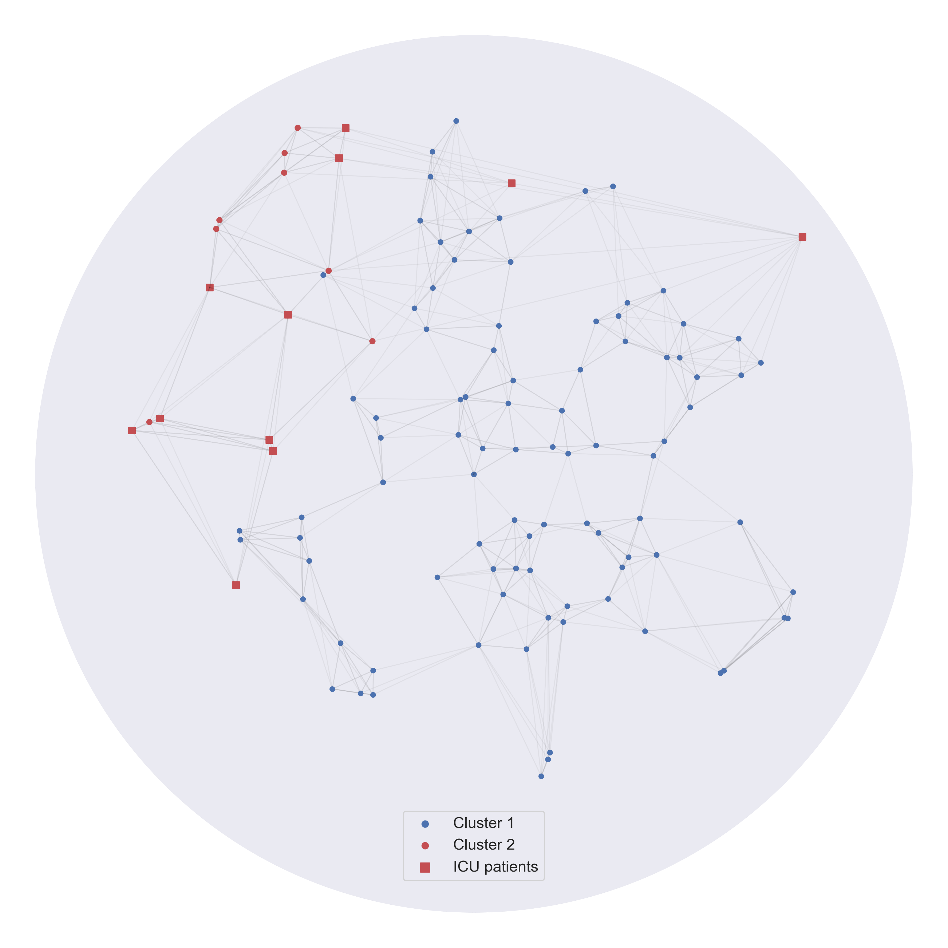

Supplement: Supplementary file 1 — Additional file 1: Fig. S1. A visualization of the clusters related to the severe malaria patients, obtained using K-means. [file 12936_2024_4869_MOESM1_ESM.tif]
